# The synergetic effect of Imipenem-clarithromycin combination in the *Mycobacteroides abscessus* complex

**DOI:** 10.1186/s12866-020-02000-5

**Published:** 2020-10-19

**Authors:** Satomi Takei, Hiroaki Ihara, Shinsaku Togo, Ayako Nakamura, Yuichi Fujimoto, Junko Watanabe, Kana Kurokawa, Kohei Shibayama, Issei Sumiyoshi, Yusuke Ochi, Moe Iwai, Takahiro Okabe, Masayoshi Chonan, Shigeki Misawa, Akimichi Ohsaka, Kazuhisa Takahashi

**Affiliations:** 1 Department of Clinical Laboratory, Juntendo University, Faculty of Medicine & Graduate School of Medicine, Tokyo, Japan; 2 Department of Respiratory Medicine, Juntendo University, Faculty of Medicine & Graduate School of Medicine, 2-1-1 Hongo, Bunkyo-Ku, Tokyo, 113-8421 Japan; 3 Koto Hospital, Tokyo, Japan; 4 Research Institute for Diseases of Old Ages, Juntendo University, Faculty of Medicine & Graduate School of Medicine, Tokyo, Japan; 5 Juntendo Tokyo Koto Geriatric Medical Center, Tokyo, Japan; 6 Leading Center for the Development and Research of Cancer Medicine, Juntendo University, Faculty of Medicine & Graduate School of Medicine, Tokyo, Japan; 7 Department of Transfusion Medicine and Stem Cell Regulation, Juntendo University, Faculty of Medicine & Graduate School of Medicine, Tokyo, Japan

**Keywords:** Clarithromycin, Fractional inhibitory concentration index, Imipenem, *Mycobacteroides abscessus*

## Abstract

**Background:**

Nontuberculous mycobacteria (NTM) are ubiquitous organisms and the incidence of NTM infections has been increasing in recent years. *Mycobacteroides abscessus* (*M. abscessus*) is one of the most antimicrobial-resistant NTM; however, no reliable antibiotic regimen can be officially advocated. We evaluated the efficacy of clarithromycin in combination with various antimicrobial agents against the *M. abscessus* complex.

**Results:**

Twenty-nine clinical strains of *M. abscessus* were isolated from various clinical samples. Of the isolates, 10 (34.5%) were of *M. abscessus* subsp. *abscessus*, 18 (62.1%) of *M. abscessus* subsp. *massiliense*, and 1 (3.4%) of *M. abscessus* subsp. *bolletii*. MICs of three antimicrobial agents (amikacin, imipenem, and moxifloxacin) were measured with or without clarithromycin. The imipenem-clarithromycin combination significantly reduced MICs compared to clarithromycin and imipenem monotherapies, including against resistant strains. The association between susceptibility of the *M. abscessus* complex and each combination of agents was significant (*p* = 0.001). Adjusted residuals indicated that the imipenem-clarithromycin combination had the synergistic effect (adjusted residual = 3.1) and suppressed the antagonistic effect (adjusted residual = − 3.1). In subspecies of *M. abscessus* complex, the association with susceptibility of *M. abscessus* subsp. *massiliense* was similarly statistically significant (*p* = 0.036: adjusted residuals of synergistic and antagonistic effect respectively: 2.6 and − 2.6). The association with susceptibility of *M. abscessus* subsp. *abscessus* also showed a similar trend but did not reach statistical significance.

**Conclusion:**

Our data suggest that the imipenem-clarithromycin combination could be the recommended therapeutic choice for the treatment of *M. abscessus* complex owing to its ability to restore antimicrobial susceptibility.

## Background

NTM are ubiquitous organisms that cause diverse types of infectious diseases in humans, including in lungs, the lymphatic system, skin, soft tissue, bone disease, and are disseminated. The morbidity of NTM has been increasing worldwide [1, 2]; the 2014 nationwide survey of NTM in Japan revealed that the incidence of pulmonary NTM (14.7 cases/100,000 person/year) has overtaken that of tuberculosis (12.9 cases/100,000 person/year) [3]. Above all, the *Mycobacterium avium* complex (88.8%) were the most frequently isolated organisms, followed by *Mycobacterium kansasii* (4.3%) and the *Mycobacteroides abscessus* complex (3.3%). Notably, the incidence of *M. abscessus*-infected pulmonary disease has dramatically increased in Japan, from 0.1 cases/100,000 person/year in 2001 to 0.5 cases/100,000 person/year in 2014. *M. abscessus* is one of the treatment-refractory NTM, characterized by rapid growth and multidrug resistance. *M. abscessus*, which is frequently isolated from respiratory tracts of patients with cystic fibrosis (CF), has been the leading cause of rapid growing mycobacteria in CF since the 2000s [4, 5]. The critical features of *M. abscessus* involve resistance to most antibiotics in clinical use, including first line antitubercular drugs [6, 7]. The 2007 American Thoracic Society/Infectious Diseases Society of America (ATS/IDSA) statement recommended multidrug therapy, including a macrolide and one or more parenteral agents (e.g., amikacin, cefoxitin, or imipenem) [8]; however, recommendations for the treatment of *M. abscessus* are known to be of limited efficacy [9]. Recently, three subspecies of *M. abscessus* have been defined: *M. abscessus* subsp. *abscessus*, *M. abscessus* subsp. *bolletii*, and *M. abscessus* subsp. *massiliense. M. abscessus* subsp. *massiliense* specifically lacks the *erm* (41) gene associated with macrolide resistance, and thus, the macrolide susceptibility among *M. abscessus* subsp. *massiliense* and *M. abscessus* subsp. *abscessus* and *bolletii* is different [10, 11]. For this reason, some experts recommend non-macrolide combinations for treatment for macrolide-resistant *M. abscessus* subspecies, based on identified in vitro susceptibilities [12]. Here, we propose new insights into the synergistic effects on *M. abscessus* susceptibility achieved in vitro by clarithromycin in combination with other antimicrobials.

## Results

### Clinical features of three subspecies of *M. abscessus* complex

Twenty-nine clinical strains of *M. abscessus* were isolated from various clinical samples at the Juntendo university hospital from 2011 to 2019. The characteristics of patients from which *M. abscessus* complex were isolated are shown in Table 1. Twenty-two of 29 (75.9%) patients were diagnosed with *M. abscessus* complex from the culturing of sputum or bronchial lavage. Of the isolates, 10 (34.5%) were of *M. abscessus* subsp. *abscessus*, 18 (62.1%) of *M. abscessus* subsp. *massiliense*, and 1 (3.4%) of *M. abscessus* subsp. *bolletii* as determined by multi-locus sequence analysis. The treatment history indicated that 24 of 29 (82.8%) patients had received antibiotics in the last 3 months, including macrolides, and 10 of 29 (34.5%) patients had received immunosuppressive treatment including corticosteroids before the culture. *erm* (41) sequevars affected clarithromycin susceptibility in *M. abscessus*, namely, *erm* (41) with a large deletion and C28 sequevar related to susceptibility, and with T28 sequevar related to resistance. All 18 strains of *M. abscessus* subsp. *massiliense* possessed *erm* (41) with a large deletion and only 2 strains of *M. abscessus* subsp. *abscessus* had erm (41) with C28 sequevar.

**Table 1:** The characteristics of patients from which *M. abscessus* complex were isolated

	*N* = 29
Sex (Male/Female)	12/17
Median age (range)	65 (38–83)
Smoking history, N (%)	9 (31.0)
*M.abscessus* complex subtype, N (%)	
*M.abscessus* subsp. *abscessus*	10 (34.5)
*M.abscessus* subsp. *masiliense*	18 (62.1)
*M.abscessus* subsp. *bolletii*	1 (3.4)
*M.abscessus* complex detected from, N (%)	
Sputum or bronchial lavage	22 (75.9)
Others	7 (24.1)
*erm* (41) gene status, N (%)	
deletion	18 (17.2)
T28	9 (31.0)
C28	2 (6.9)
Pretreatment of antibiotics within 3 months, N (%)	
Macrolides	5 (17.2)
Fluoroquinolones	5 (17.2)
Tetracyclines	2 (6.9)
Others	12 (41.4)
Comorbidity, N (%)	
Bronchiectasis	10 (34.5)
Diabetes mellitus	4 (13.8)
Immunodeficiency (non HIV)	2 (6.9)
Malignancy	7 (24.1)
Concomitant medications, N (%)	
Corticosteroids	6 (20.7)
Immunosupressant	4 (13.8)

*Abbreviations*: *HIV* human immunodeficiency virus

### Susceptibility to antimicrobial agents in combination with clarithromycin

**Fig. 1:**
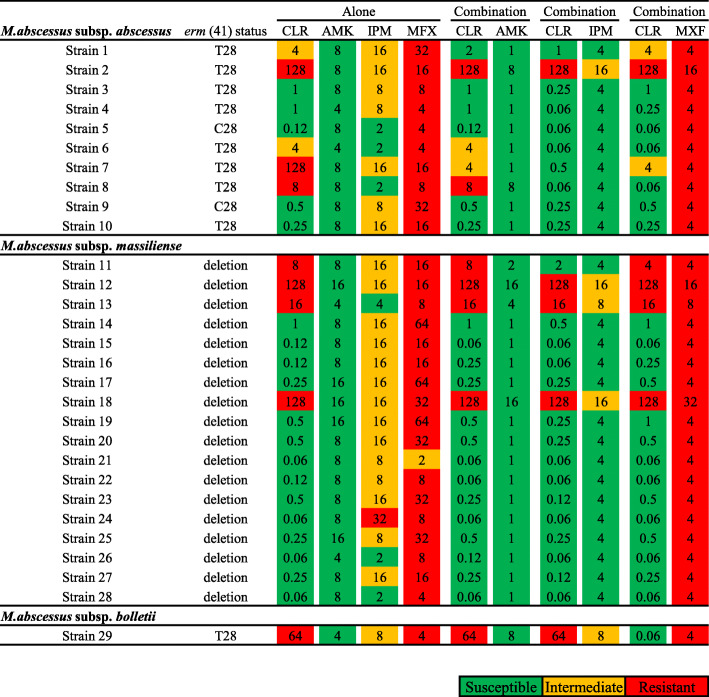
MIC distributions for amikacin, imipenem, and moxifloxacin combined with clarithromycin, categorized into three subspecies of *M. abscessus* complex on day 7. Green color indicates susceptibility, yellow color indicates intermediate, and red color indicates resistance to *M. abscessus*. Abbreviations: CLR, clarithromycin; AMK, amikacin; IPM, imipenem; MXF, moxifloxacin

MICs on day 3, 7, and 14 were recorded (Fig. 1, S1, and S2). Bacterial growth on day 3 was insufficient. MIC on day 14 was equivalent as compared to MICs on day 7; however, over-growth was observed after 14-day incubation. Thus, MICs on day 7 were used in following experiments. The susceptibility to a combination of clarithromycin and antimicrobial agents was compared to that of the antimicrobial agents alone, categorized into each subspecies of *M. abscessus* complex (Fig. 1). The MICs of three antimicrobial agents (amikacin, imipenem, and moxifloxacin) were measured with or without clarithromycin. Notably, the use of imipenem-clarithromycin combination mostly reduced the MIC of imipenem. The combination also reduced the MIC of clarithromycin, even in clarithromycin-resistant subspecies of *M. abscessus* complex. The effect of reducing clarithromycin MICs by the imipenem-clarithromycin combination was stronger than that of the amikacin- and moxifloxacin-clarithromycin combination. In 1 strain of *M. abscessus* subsp. *abscessus* and 3 strains of *M. abscessus* subsp. *massiliense*, susceptibility was not restored by the combined use of clarithromycin and imipenem, and only 1 strain of *M. abscessus* subsp. *bolletii* did not respond to any combination. MICs of clarithromycin alone in *M. abscesssus* complex with a large deletion and C28 sequevar in *erm* (41) were significantly lower than that of T28 sequevar (*p* = 0.0075). In subspecies of *M. abscessus* complex, MICs of imipenem and clarithromycin in combination were significantly less than that of either clarithromycin or imipenem alone in both *M. abscessus* subsp. *massiliense* and *abscessus* (*p* < 0.001 for both subspecies) (Table S1).

**Fig. 2:**
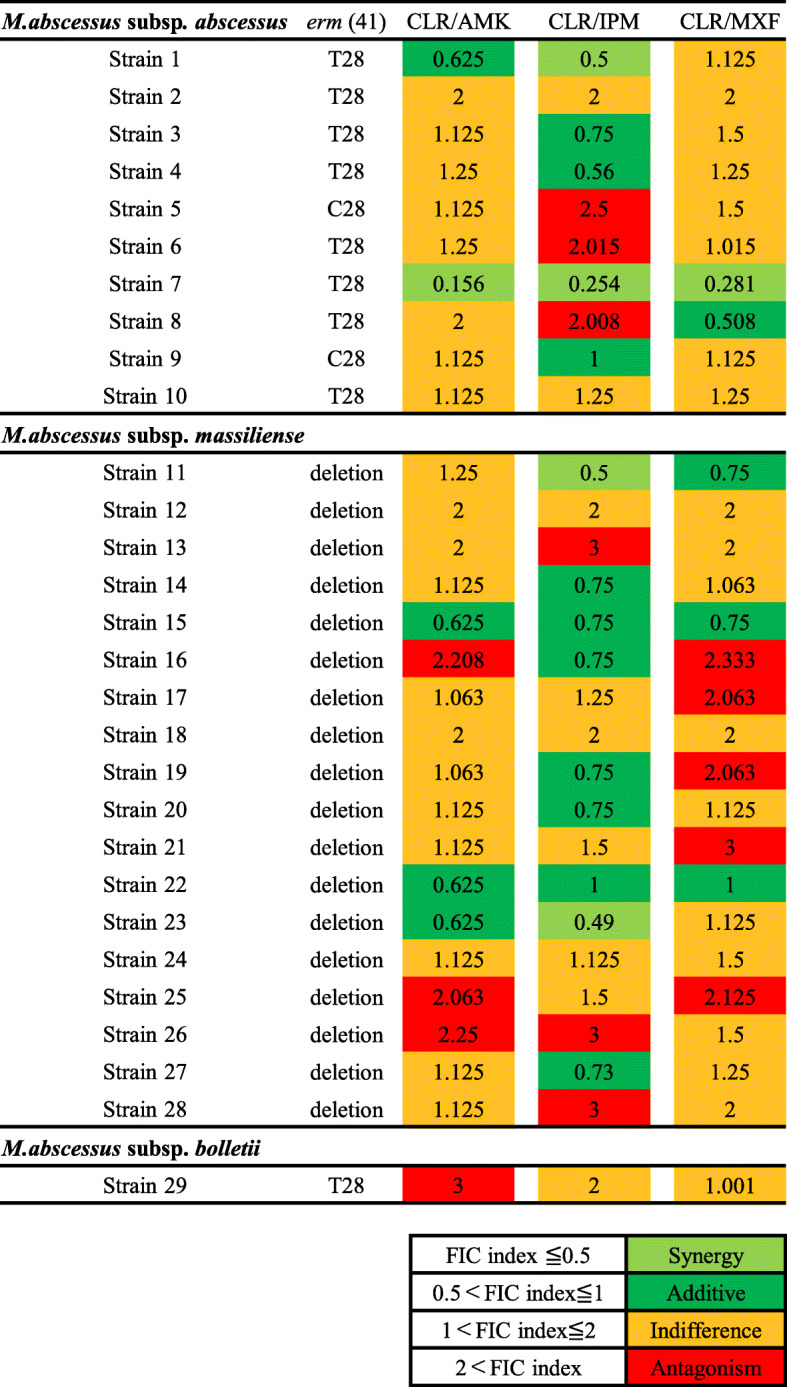
FIC index of amikacin, imipenem, and moxifloxacin combined with clarithromycin, categorized into three subspecies of *M. abscessus*. Light green color indicates synergy, green color indicates additive, yellow color indicates indifference, and red color indicates antagonism in each combination. Abbreviations: CLR, clarithromycin; AMK, amikacin; IPM, imipenem; MXF, moxifloxacin; FIC index, fractional inhibitory concentration index

We next determined the synergistic effect of the imipenem-clarithromycin combination as compared to amikacin- or moxifloxacin-clarithromycin combinations, using the fractional inhibitory concentration (FIC) index as described in previous paper [13] (Fig. 2). Susceptibility was divided into two classes, synergy and additive as a synergistic effect and indifference and antagonism as an antagonistic effect. Table 2 showed the number of isolates showing synergistic and antagonistic effects with each combination. In *M. abscessus* complex, 14 strains (48.3%) revealed synergistic effects for imipenem-clarithromycin combination, in contrast, only 5 strains (17.2%) for moxifloxacin- or amikacin-clarithromycin combination. In *M. abscessus* subsp. *massiliense,* 9 strains (50.0%) revealed synergistic effects for imipenem-clarithromycin combination, only 3 strains (16.7%) for moxifloxacin- or amikacin-clarithromycin combination. In *M. abscessus* subsp. *abscessus,* 5 strains (50.0%) revealed synergistic effects for imipenem-clarithromycin combination, only 2 strains (20.0%) for moxifloxacin- or amikacin-clarithromycin combination.

**Table 2:** The number of synergistic and antagonistic combination with clarithromycin and each antimicrobial

		CLR/AMK	CLR/IPM	CLR/MXF	
Species	Categories of FIC index	N (%, Adjusted residual)			*p* value
*M.abscessus* complex	Synergy + Additive	5 (17.2, − 1.5)	14 (48.3, 3.1**)	5 (17.2, − 1.5)	0.001**
N = 29†	Indifference + Antagonism	24 (82.8, 1.5)	15 (51.7, − 3.1**)	24 (82.8, 1.5)	
*M. abscessus* subsp. *massiliense*	Synergy + Additive	3 (16.7, −1.3)	9 (50.0, 2.6**)	3 (16.7, − 1.3)	0.036*
*N* = 18	Indifference + Antagonism	15 (83.3, 1.3)	9 (50.0, −2.6**)	15 (83.3, 1.3)	
*M. abscessus* subsp. *abscessus*	Synergy + Additive	2 (20.0, −0.8)	5 (50.0, 1.7)	2 (20.0, −0.8)	0.24
*N* = 10	Indifference + Antagonism	8 (80.0, 0.8)	5 (50.0, −1.7)	8 (80.0, 0.8)	

† including *M. abscessus* subsp. *boletii* (n = 1)

* *p* value < 0.05, ** *p* value < 0.01

* adjusted residuals > |1.96|, ** adjusted residuals > |2.58|

*Abbreviations*: *FIC index* fractional inhibitory concentration index, *CLR* clarithromycin, *AMK* amikacin, *IPM* imipenem, *MXF* moxifloxacin

### Association of clinical features with susceptibilities to the imipenem-clarithromycin combination

We investigated whether susceptibility to the imipenem-clarithromycin combination might associate with clinical status. The isolates from patients with immunosuppression and/or administered immunosuppressive drugs and/or corticosteroids revealed synergistic effects rather than antagonistic effects (*p* = 0.040) (Table 3). The other clinical parameters such as age, sex, smoking history, bronchiectasis lesion, a treatment history of antibiotics, and *erm* (41) gene status did not influence the effects of imipenem-clarithromycin combination.

**Table 3:** The number of synergistic and antagonistic combination with clarithromycin and imipenem in each clinical status

	FIC index		
	Synergy + Additive *N* = 14 (%)	Indifference + Antagonism *N* = 15 (%)	*p* value
Age			
< 65 years	7 (24.1)	7 (24.1)	0.86
≥65 years	7 (24.1)	8 (27.6)	
Sex			
Male	6 (20.7)	6 (20.7)	0.88
Female	8 (27.6)	9 (31.0)	
Smoking history			
Yes	3 (10.3)	6 (20.7)	0.43
No	11 (37.9)	9 (31.0)	
With bronchiectasis			
Yes	4 (13.8)	6 (20.7)	0.70
No	10 (34.5)	9 (31.0)	
With immunosuppression			
Yes	10 (34.5)	5 (17.2)	0.040*
No	4 (13.8)	10 (34.5)	
Pretreatment of antibiotics			
Yes	6 (20.7)	8 (27.6)	0.57
No	8 (27.6)	7 (24.1)	
*erm* (41) gene status			
del + C28	10 (34.5)	10 (34.5)	0.78
T28	4 (13.8)	5 (17.2)	

Antibiotics including clarithromycin (*n* = 3)

*p* value < 0.05, ** *p* value < 0.01

*Abbreviations*: *FIC index* fractional inhibitory concentration index, *del* deletion

## Discussion

We demonstrated here that the MICs of clarithromycin and imipenem were significantly reduced by the administration of an imipenem-clarithromycin combination. We propose a new therapeutic benefit by which the imipenem-clarithromycin combination could reduce the MICs of *M. abscessus* isolates showing resistance to clarithromycin and/or imipenem. The isolates included *M. abscessus* subsp. *abscessus,* well known among the three subspecies to show high resistance rate to macrolides [10, 11]. Furthermore, this combination may be suitable for treatment of *M. abscessus* complex in patients with immunosuppression.

There were several problems involved in the current recommended treatment for *M. abscessus*, due to the lack of clinical outcomes, and uncertain interactions present in multidrug combination therapy; thus, there is still limited reliable evidence to promote a global standard treatment regimen for the three subspecies of *M. abscessus* complex. Previous in vitro studies have demonstrated that treatment with the standard regimen therapy (combinations of clarithromycin, amikacin, and cefoxitin) failed to effectively inhibit the growth of *M. abscessus* due to acquired drug resistance [14]. In vivo, the triple-drug regimen was equally or less effective against *M. abscessus* than cefoxitin alone [15]. A systematic review revealed different outcomes of macrolide-containing combination regimens against *M. abscessus* subsp. *abscessus* and *massiliense.* Macrolide-containing combination regimens for *M. abscessus* subsp. *abscessus* induced lower rates of negative conversion of sputum culture and higher recurrence rates than that of *M. abscessus* subsp. *massiliense* [16]. For these reasons, the appropriate drug therapy against *M. abscessus* remains uncertain. *M. abscessus* complex spontaneously produce broad-spectrum β-lactamases, resulting in reduced susceptibility to β-lactams, including imipenem. The combination therapy of imipenem with rifabutin or amikacin was more effective than the monotherapy of imipenem against *M. abscessus* complex [17, 18]. Miyasaka et al. verified the best combined antibiotics with imipenem and described that the imipenem-clarithromycin combination had a high rate of synergistic and additive effects, and revealed a decrease in the MIC values inhibiting 50% or 90% of *M. abscessus* complex [19]. Interestingly, we checked the effect of clarithromycin synergy with each antibiotic, resulting in the same combination therapy. Further, our data revealed the details of Miyasaka’s findings to evaluate the effect of imipenem-clarithromycin combination in each subspecies or patient character. Although the exact mechanism for the synergistic effect of clarithromycin combinations was unknown. Therefore, imipenem may be useful in combination with clarithromycin for the treatment of *M. abscessus* complex. Limitations of the present study include the lack of clinical outcomes measured in patients with *M. abscessus* complex treated with imipenem-clarithromycin combination therapy. The number of clinical isolates used in the study was still insufficient when we separately analyzed the susceptibility of *M. abscessus* subspecies. Therefore, we could not demonstrate the synergistic effects of imipenem-clarithromycin combination in *M. abscessus* subsp. *abscessus*. Further experiments were required to confirm the efficacy of the combination regimen.

## Conclusion

In our in vitro study, we demonstrated the synergistic effect of the imipenem-clarithromycin combination in restoring *M. abscessus* complex antimicrobial susceptibility. Further, this synergistic effect may occur not only in *M. abscessus* subsp. *massiliense*, but also in *M. abscessus* subsp. *abscessus*. Thus, our present results suggest that the imipenem-clarithromycin combination could be an effective treatment regimen against both *M. abscessus* subsp. *massiliense* and *M. abscessus* subsp. *abscessus*.

## Methods

### Determination of *M. abscessus* complex

All material samples suspected of mycobacterial contamination in the Juntendo university hospital were cultured in mycobacteria growth indicator tube (MGIT; Becton Dickinson, USA) broth and incubated at 37 °C in the BACTEC MGIT 960 (Becton Dickinson, USA) instrument with ambient air. MGIT positive tubes were classified as *M. abscessus* based on the results of DNA–DNA hybridization (DDH) analysis (DDH Mycobacterium “Kyokuto” kit; Kyokuto Pharmaceutical Industrial, Japan) or matrix-assisted laser-desorption/ionization time-of-flight mass spectrometry (MALDI-TOF MS). Detected species were reconfirmed as three subspecies of *M. abscessus* complex by sequencing the *16S rRNA*, *rpoB*, *hsp65*, and *erm* genes [20, 21]. All strains of *M. abscessus* were cultured on BD trypticase soy agar II with 5% sheep blood (Blood agar; Nippon Becton-Dickinson and Company, Japan) at 35 °C for approximately 4 to 6 days in an aerobic atmosphere. The study protocol was approved by the Ethics Committee of Juntendo University School of Medicine (no. 18–010 and 19–038).

### MALDI-TOF MS analysis

MALDI-TOF MS analysis was performed based on previously described methods [22]. Colonies of *M. abscessus* complex on blood agar were scratched with a needle, and particles on the needle surface were diluted in 50 μL 80% trifluoroacetic acid. After incubation for 15 min at room temperature, the solution was added to 150 μL distilled water and 200 μL 100% acetonitrile, followed by a centrifugation step (16,200×g, 2 min). One microliter of the cleared supernatant containing the bacterial extract was transferred onto a MALDI target plate (Bruker Daltonik, Germany). We overlaid dried spots with MALDI matrix (10 mg/mL α-cyano-4-hydroxy-cinnamic acid [α-HCCA] in 50% acetonitrile:2.5% trifluoroacetic acid) (Bruker Daltonik, Germany). After drying of the matrix, we conducted MALDI-TOF MS analysis with a Microflex LT/SH benchtop mass spectrometer (Bruker Daltonik, Germany) equipped with a 60-Hz nitrogen laser. We had optimized parameter settings (ion source 1 [IS1], 20 kV; IS2, 18.2 kV; lens, 6.85 kV; detector gain, 2854 V; gating, none) for the mass range between 2000 and 20,000 Da. We achieved spectra in the positive linear mode with the maximum laser frequency. An external standard (bacterial test standard [BTS]) (Bruker Daltonik, Germany) was applied for instrument calibration. Data evaluation was performed by visually comparing spectra to search for peak shifts using flexAnalysis 3.4 (Bruker Daltonik, Germany).

### PCR amplification and DNA sequencing

DNA was extracted from cultured colonies using the DNeasy UltraClean Microbial Kit (QIAGEN, Germany), and PCR was conducted using Ex Taq DNA polymerase, hot-start version (Takara, Japan) according to the manufacturer’s instructions. The gene-specific primer pairs used for PCR analysis are listed in Table 4; these primers were used in previous studies [23, 24]. The sequencing PCR products were purified with the BigDye XTerminator purification kit (Life Technologies, USA) and samples were loaded on the ABI Prism 3130 Genetic Analyzer (Thermo Fisher Scientific, USA). The DNA sequencing results were analyzed using a BLAST search to identify sequence similarity between samples and the three species of *M. abscessus* complex.

**Table 4:** Forward and backward primers used for PCR

Target	Sequence
*16S rRNA*	Forward, 5′-AGA GTT TGA TCM TGG CTC AG-3′ Reverse, 5′-TAC GGT TAC CTT GTT ACG AC-3′
*rpoB*	Forward, 5′-GAG GGT CAG ACC ACG ATG AC −3′ Reverse, 5′-AGC CGA TCA GAC CGA TGT T-3′
*hsp65*	Forward, 5’ACC AAC GAT GGT GTG TCC AT − 3′ Reverse, 5′ CTT GTC GAA CCG CAT ACC CT-3′
*erm*	Forward, 5′-GAC CGG GCC TTC GTG AT − 3′ Reverse, 5′-GAC TTC CCC GCA CCG ATT CC-3′

### Antimicrobial susceptibility testing

Susceptibility testing was performed according to Clinical and Laboratory Standard Institute (CLSI) guideline M24-A2 [25]. MIC determinations and synergy testing were performed by the checkerboard method using frozen broth microdilution plates (Eiken Chemical Co., Ltd., Japan). The ranges of antibiotic concentrations tested were as follows: amikacin (AMK) 0.25 to 64 μg/mL, clarithromycin (CLR) 0.06 to 64 μg/mL, imipenem (IPM) 4 to 32 μg/mL, and moxifloxacin (MXF) 1 to 32 μg/mL. MICs of each antimicrobial agent were determined by broth microdilution methods as recommended by the CLSI. The panels were prepared with a 96-channel dispenser and stored at − 80 °C until use. CLR were dispensed alone in the first row, and IPM, AMK, or MXF were dispensed in the first column. Each well was inoculated with a concentration of 1 × 10^5^ colony-forming units (CFU)/mL. The MICs were determined after 3, 7, 14 days of incubation at 35 °C. The MIC breakpoints, indicating susceptible, intermediate, and resistant strains, were interpreted according to the CLSI criteria for amikacin, cefoxitin, ciprofloxacin, clarithromycin, doxycycline, imipenem, linezolid, moxifloxacin, trimethoprim/sulfamethoxazole, and tobramycin (Table 5) [25]. The effect of each agent combined with clarithromycin was evaluated using FIC index analysis [13].

**Table 5:** Antimicrobial agents and MIC breakpoints for rapidly growing mycobacteria

	MIC (μg/mL) for category		
Antimicrobial agents	Susceptible	Intermediate	Resistant
Amikacin	≤16	32	≥64
Cefoxitin	≤16	32–64	≥128
Ciprofloxacin	≤1	2	≥4
Clarithromycin	≤2	4	≥8
Doxycycline	≤1	2–4	≥8
Imipenem	≤4	8–16	≥32
Linezolid	≤8	16	≥32
Moxifloxacin	≤1	2	≥4
Trimethoprim- sulfamethoxazole	≤ 2/38	–	≥ 4/76
Tobramycin	≤2	4	≥8

### Statistical analysis

Categorical variables were compared using the chi-square test or Fisher’s exact test. The evaluation of changes in MIC was performed using the Wilcoxon signed-rank test. Differences were considered significant at *p* < 0.05. When the chi-square test results were statistically significant, adjusted residuals were calculated to determine which particular associations were significant. Adjusted residuals were significant at *p* < 0.05 level if they were less than − 1.96 or more than 1.96, and were significant at *p* < 0.01 level if they were less than − 2.58 or more than 2.58. All statistical analyses were performed using the SPSS software program (version 20, IBM Japan, Japan).

## Supplementary information

**Additional file 1: Supplementary figure 1**. MIC distributions for amikacin, imipenem, and moxifloxacin combined with clarithromycin, categorized into three subspecies of *M. abscessus* complex on day 3. Green color indicates susceptibility, yellow color indicates intermediate, and red color indicates resistance to *M. abscessus*. Abbreviations: CLR, clarithromycin; AMK, amikacin; IPM, imipenem; MXF, moxifloxacin; NA, not assessed. **Supplementary figure 2**. MIC distributions for amikacin, imipenem, and moxifloxacin combined with clarithromycin, categorized into three subspecies of *M. abscessus* complex on day 14. Green color indicates susceptibility, yellow color indicates intermediate, and red color indicates resistance to *M. abscessus*. Abbreviations: CLR, clarithromycin; AMK, amikacin; IPM, imipenem; MXF, moxifloxacin. **Supplementary Table 1**. The changes of median MIC of clarithromycin and imipenem between monotherapy and combination therapy.

## Data Availability

The datasets during the current study available from the corresponding author on reasonable request.

## Abbreviations

NTM: Nontuberculous mycobacteria
*M. abscessus*: *Mycobacteroides abscessus*
CF: Cystic fibrosis
ATS/IDSA: American Thoracic Society/Infectious Diseases Society of America
FIC: Fractional inhibitory concentration
MGIT: Mycobacteria growth indicator tube
DDH: DNA–DNA hybridization
MALDI-TOF MS: Matrix-assisted laser-desorption/ionization time-of-flight mass spectrometry
BTS: Bacterial test standard
CLSI: CLINICAL and Laboratory Standard Institute
AMK: Amikacin
CLR: Clarithromycin
IPM: Imipenem
MXF: Moxifloxacin
HIV: Human immunodeficiency virus
CFU: Colony-forming units
