# Supplementary material for: The synergetic effect of Imipenem-clarithromycin combination in the Mycobacteroides abscessus complex
Source: BMC Microbiol. 2020 Oct 19;20:316. doi: 10.1186/s12866-020-02000-5 (PMC7574464; doi:10.1186/s12866-020-02000-5)
Supplement: Supplementary file 1 — Additional file 1: Supplementary figure 1. MIC distributions for amikacin, imipenem, and moxifloxacin combined with clarithromycin, categorized into three subspecies of M. abscessus complex on day 3. Green color indicates susceptibility, yellow color indicates intermediate, and red color indicates resistance to M. abscessus. Abbreviations: CLR, clarithromycin; AMK, amikacin; IPM, imipenem; MXF, moxifloxacin; NA, not assessed. Supplementary figure 2. MIC distributions for amikacin, imipenem, and moxifloxacin combined with clarithromycin, categorized into three subspecies of M. abscessus complex on day 14. Green color indicates susceptibility, yellow color indicates intermediate, and red color indicates resistance to M. abscessus. Abbreviations: CLR, clarithromycin; AMK, amikacin; IPM, imipenem; MXF, moxifloxacin. Supplementary Table 1. The changes of median MIC of clarithromycin and imipenem between monotherapy and combination therapy. [file 12866_2020_2000_MOESM1_ESM.docx]

**Supplementary figure**

**Supplementary figure 1**: MIC distributions for amikacin, imipenem, and moxifloxacin combined with clarithromycin, categorized into three subspecies of *M. abscessus* complex on day 3. Green color indicates susceptibility, yellow color indicates intermediate, and red color indicates resistance to *M. abscessus*. Abbreviations: CLR, clarithromycin; AMK, amikacin; IPM, imipenem; MXF, moxifloxacin; NA, not assessed.


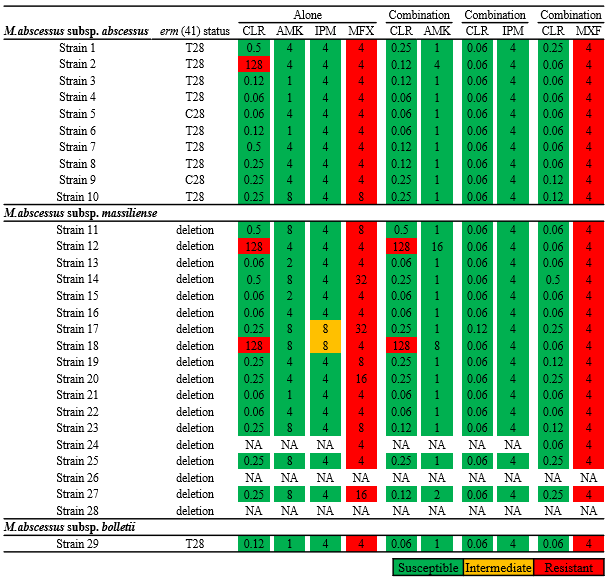


**Supplementary figure 2**: MIC distributions for amikacin, imipenem, and moxifloxacin combined with clarithromycin, categorized into three subspecies of *M. abscessus* complex on day 14. Green color indicates susceptibility, yellow color indicates intermediate, and red color indicates resistance to *M. abscessus*. Abbreviations: CLR, clarithromycin; AMK, amikacin; IPM, imipenem; MXF, moxifloxacin.


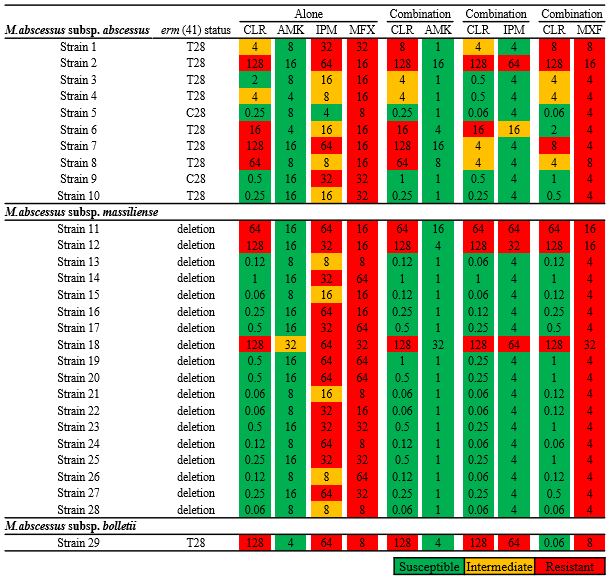


**Supplementary Table**

**Supplementary Table 1**: The changes of median MIC of clarithromycin and imipenem between monotherapy and combination therapy.

| Species | Median MIC of CLR (monotherapy) | Median MIC of CLR (combination therapy) | *p* value | Median MIC of IPM (monotherapy) | Median MIC of IPM (combination therapy) | *p* value |
| --- | --- | --- | --- | --- | --- | --- |
| *M.abscessus* complex | 0.5 | 0.25 | <0.0001** | 16 | 4 | <0.001** |
| N=29† |  |  |  |  |  |  |
| *M. abscessus* subsp. *massiliense* | 2.5 | 0.25 | 0.0039** | 8 | 4 | <0.001** |
| N=18 |  |  |  |  |  |  |
| *M. abscessus* subsp. *abscessus* | 0.25 | 0.185 | 0.0078** | 16 | 4 | 0.043* |
| N=10 |  |  |  |  |  |  |

†Including *M. abscessus* subsp. *boletii* (n=1)

**p* value <0.05, ** *p* value <0.01

Abbreviations: CLR, clarithromycin; IPM, imipenem.
